# The Effect of a Transcranial Channel as a Skull/Brain Interface in High-Definition Transcranial Direct Current Stimulation—A Computational Study

**DOI:** 10.1038/srep40612

**Published:** 2017-01-13

**Authors:** Hyeon Seo, Hyoung-Ihl Kim, Sung Chan Jun

**Affiliations:** 1 Gwangju Institute of Science & Technology, School of Electrical Engineering and Computer Science, Gwangju, 61005, South Korea; 2 Gwangju Institute of Science & Technology, Department of Biomedical Science and Engineering, Gwangju, 61005, South Korea

## Abstract

A transcranial channel is an interface between the skull and brain; it consists of a biocompatible and highly conductive material that helps convey the current induced by transcranial direct current stimulation (tDCS) to the target area. However, it has been proposed only conceptually, and there has been no concrete study of its efficacy. In this work, we conducted a computational investigation of this conceptual transcranial model with high-definition tDCS, inducing focalized neuromodulation to determine whether inclusion of a transcranial channel performs effectively. To do so, we constructed an anatomically realistic head model and compartmental pyramidal neuronal models. We analyzed membrane polarization by extracellular stimulation and found that the inclusion of a transcranial channel induced polarization at the target area 11 times greater than conventional HD-tDCS without the transcranial channel. Furthermore, the stimulation effect of the transcranial channel persisted up to approximately 80%, even when the stimulus electrodes were displaced approximately 5 mm from the target area. We investigated the efficacy of the transcranial channel and found that greatly improved stimulation intensity and focality may be achieved. Thus, the use of these channels may be promising for clinical treatment.

Electrical brain stimulation is a promising method with which to modulate neural excitability and the approach can be categorized as either invasive or non-invasive, depending upon whether incisions or insertions are required. Implantable cortical stimulation has been proven to produce highly accurate excitability of neural tissue because of its invasiveness; however, this method is not as safe as those^1^,^2^,^3^ allowing neuromodulation without surgical procedures^4^,^5^,^6^,^7^. One form of noninvasive electrotherapy is high-definition tDCS (HD-tDCS), which induces its maximum stimulus-induced electric field (EF) directly beneath the stimulus electrode; however, because skull dispersion shunts away most of the current^1^,^8^,^9^,^10^, it conveys a weak current that is distributed broadly over a relatively larger area of the brain compared to invasive approaches.

To achieve intense and focused neuromodulation with minimal invasiveness, Wingeier *et al*. introduced a transcranial channel combined with tDCS^11^. A transcranial channel acts as an interface through the thickness of the skull to deliver current induced by tDCS into the targeted area. Implanted in the patient’s skull, it is made of biocompatible and highly conductive materials. To lower the risk of infection, it does not breach the extradural space. It has various designs, including an I-shaped cylindrical channel (shaft) and a T-shaped channel (shaft with hat), among others. However, this transcranial channel has only been proposed conceptually, and to date there has been no concrete study of its efficacy. Thus, it remains unknown how well a transcranial channel improves the efficiency of brain stimulation in the underlying cellular mechanisms.

In this study, we investigated the effects of a transcranial channel by combining HD-tDCS, using a computational study that allowed a cost-effective prediction of the influence of brain stimulation^6^,^12^,^13^,^14^,^15^,^16^. The spatial extent of stimulus-induced EF has been estimated in computational studies with the assumption that the EF magnitude is correlated with the modulation of cortical excitability^4^,^8^,^10^,^17^,^18^. However, it cannot reflect stimulus-evoked neuronal polarization, which is dependent on target neuronal morphology, location, and EF orientation^19^,^20^,^21^. We incorporated compartmental models of pyramidal neurons (PNs) into an anatomically realistic volume conduction head model, and then investigated the way in which implantation of a transcranial channel modulated cortical excitability. We focused on stimulus-induced membrane polarization in two kinds of pyramidal neurons (PN), layer 5 (L5) and layer 3 (L3).

Our objective was to investigate the influence of the inclusion of a transcranial channel, its shape (I- or T-shaped), and displacement of the tDCS electrode relative to the transcranial channel. This study may be the first step in assessing the use of a transcranial channel combined with HD-tDCS, which may confirm its efficacy for clinical use.

## Results

### Effects of varying the dimension of the transcranial channel

For each configuration of the transcranial channel, we calculated the EFs induced and the membrane polarizations in the brain. The shaft diameter of the I- and T-shaped channels increased from 1 to 9 mm. First, we calculated the peaks of induced EF (Table 1) and found that implantation of the transcranial channel induced 3.3 to 10.3 times greater peak EFs than did the conventional HD-tDCS without the transcranial channel. As the diameter of the shaft increased, the stimulus-induced peak EF increased in diameter by up to 7 mm; however, we observed reduced peak EFs in both the T- and I-shaped models with a shaft diameter of 9 mm. Similar peak EFs occurred in both types of transcranial channels, although the T-shaped channel induced slightly higher EFs than the I-shaped channel.

Second, in order to measure the relative focality (Table 1), we calculated the volume of the cortex, where EFs were greater than 50%, 60%, 70%, or 80% of the peak EF values, respectively. The area with the highest EF magnitude (Vol_80_) was smaller in the conventional HD-tDCS without the transcranial channel compared to that of the transcranial channel. Higher EF (Vol_50–70_) was distributed more widely in the conventional HD-tDCS, while the transcranial channel helped capture it. Thus, the transcranial channel resulted in higher relative focality than did the conventional HD-tDCS, with the smaller area higher than 50% to 70% of the peak EF. Within the HD-tDCS configurations with the transcranial channel, the relative focality decreased as the diameter of the shaft increased, because the larger area of the transcranial channel conveys the stimulus-induced EF to the extended target area.

**Figure 1:**
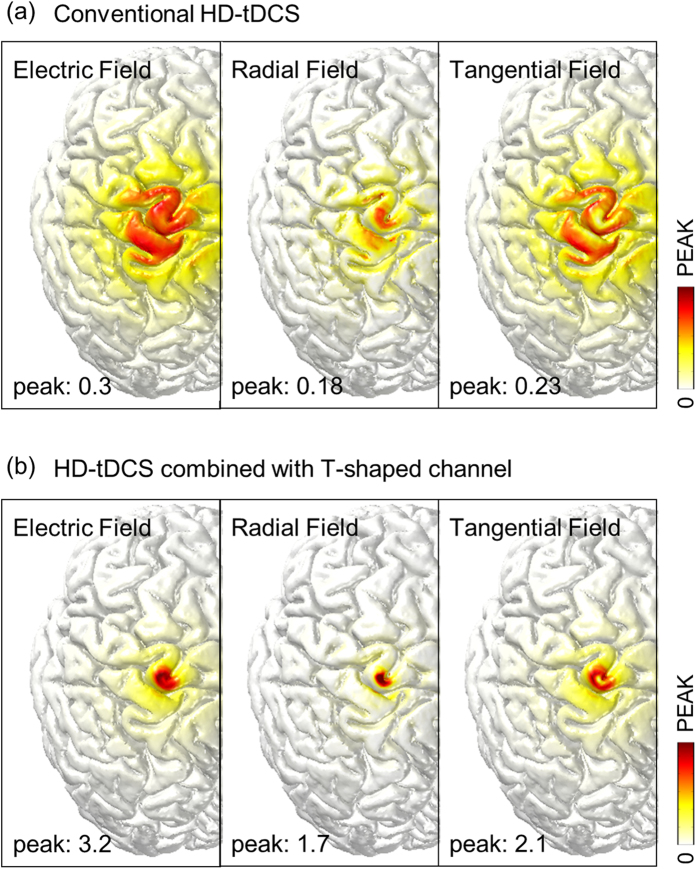
Distribution patterns of stimulus-induced EFs decomposed into radial and tangential fields on the cortical surface. Comparison of the conventional HD-tDCS without the transcranial channel (**a**) and with implantations of the T- shaped transcranial channels at the channel diameter of 7 mm (**b**); the color density scale is adjusted according to the maximum value of the EFs.

To further assess the way in which the transcranial channel improved the efficacy of stimulation, we presented spatial distributions of EFs between the conventional HD-tDCS with and without the T-shaped transcranial channel and set the channel diameter to 7 mm as it showed maximum peak EF. We focused the T-shaped channel due to I- and T-shaped channels showed comparable distributions of EFs (see Supplementary Fig. S1). As shown in Fig. 1, EF distributions depended upon whether or not the inclusion of the transcranial channel was considered, and which type of channel was incorporated. The conventional HD-tDCS resulted in a 0.3 V/m peak with an extensive EF distribution from the pre- to post-central gyri, while the inclusion of the transcranial channel increased peak EFs focused on the hand knob 10.3 fold in the T-shaped channels. In addition to EFs, the magnitudes of tangential (parallel to the cortical surface) and radial (perpendicular to the cortical surface) components of EFs are visualized (2nd and 3rd columns in Fig. 1). Tangential component was estimated by the cross product of normal vector of the cortical surface and the EFs; radial component was estimated by the dot product of normal vector and EFs. We found that radial field was highest in the top of hand knob where is directly under the active electrode, whereas the highest values of tangential field appeared toward the sulcus.

**Figure 2:**
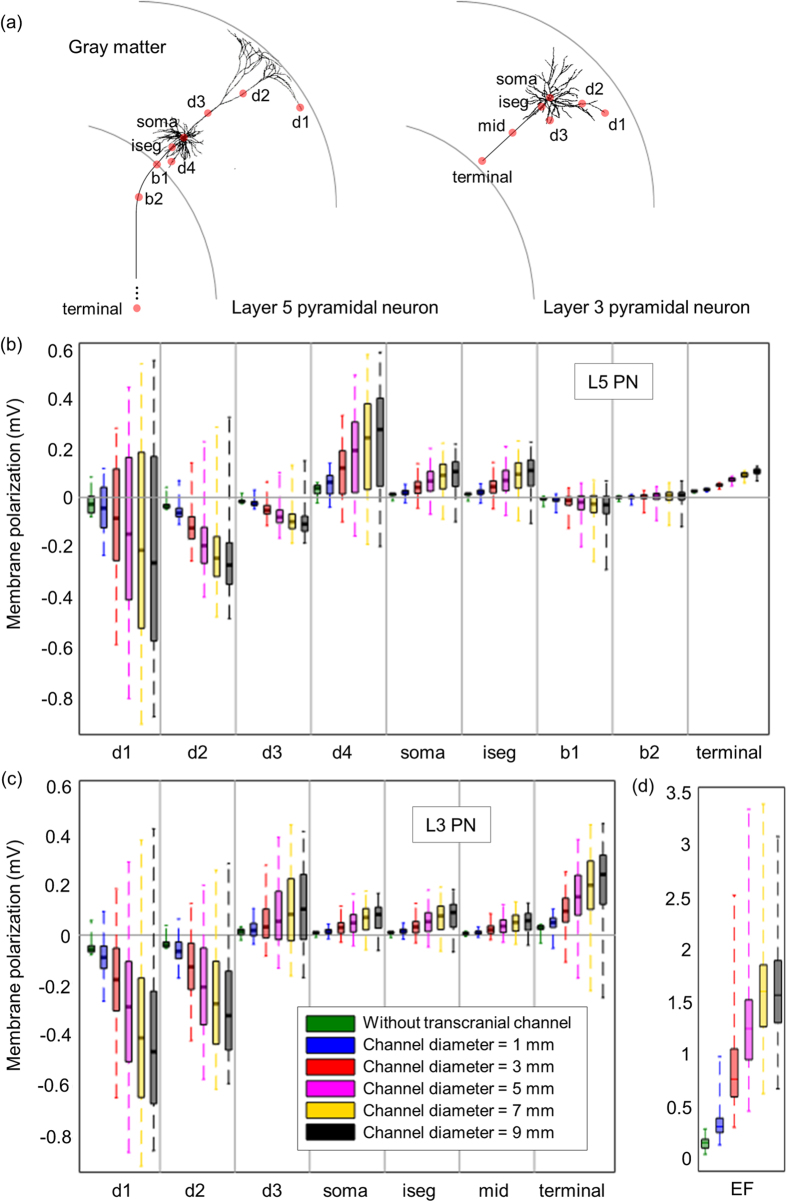
Membrane polarization for neuronal compartments in L5 and L3 PNs with various diameters of the T-shaped transcranial channel. To investigate the impact of focal and intense stimulation, we focused membrane polarizations of PNs in the region of interest (ROI) with 5 mm radius in the middle of the hand knob, where it is directly under the center electrode, and thus total 112 numbers of L5 and L3 PNs were analyzed. Membrane potentials are examined in each compartment and indicated by red dots on the neuronal morphology (**a**); in L5 PNs, we simulated membrane potentials at four dendrites (d1, d2, d3, and d4), the soma, initial segment (iseg), axon at the boundary between GM and WM (b1), bent area (b2), and terminal. In L3 PNs, we examined three dendrites (d1, d2, and d3), the soma, initial segment (iseg), middle (mid), and the terminal part of the axon. We then analyzed the membrane polarization (**b** and **c**) and induced EF (**d**) within the given ROI by the median and the first to third quartiles spanning the central rectangles, and whiskers indicate maximum and minimum values.

The excitability of the L5 and L3 PNs was then simulated in the region of interest (ROI) with 5 mm radius in the middle of the hand knob, where the top of the precentral gyrus is directly under the active electrode, over varying transcranial channel diameters. We analyzed the distributions of membrane polarization and induced EF in ROI. Note that the electric field flowed directed inward to the ROI, because ROI was a small area and located directly under the active electrode. Figure 2 shows simulated steady-state membrane potentials of L5 and L3 PNs, which include the soma, axon initial segment, bend, terminal, node crossing the gray matter (GM) and white matter (WM) boundary, and some dendrite points. PNs with a non-symmetric dendritic morphology were modulated by the polarity and orientation of the EF, and thus dendrites might have complex polarization patterns^20^,^22^,^23^. In this study, we identified some dendritic trees that suggest membrane depolarization or hyperpolarization according to their position. We found that the maximum polarization increased in the upper part of the dendrites and axon terminal as the channel diameter increased, and that the rate of increase in polarization with increasing channel diameter decreased, so that polarization at the channel diameter of 9 mm was comparable to that at the diameter of 7 mm compared to other configurations. However, the somatic polarization and other compartments show a similar tendency in peak EF behavior, i.e., they increased marginally by up to 7 mm and then decreased slightly at the 9 mm diameter. Overall, as we observed in previous results, the I-shaped transcranial channel (not shown) shows a comparable and slightly lower mean and maximum value of polarization than did the T-shaped channel. Generally, the dendrites’ upper parts were hyperpolarized, and the soma’s lower parts were depolarized; the transcranial channel amplified this polarization. The peak somatic polarization in L5 PNs for the transcranial channel with 7 mm shaft diameter (that induced maximum somatic polarization) was approximately 11 times higher than without the transcranial channel, and, similarly, we observed a 12-fold increased peak somatic polarization in the L3 PNs when the transcranial channel was incorporated. A smaller deviation was observed in the conventional HD-tDCS without the transcranial channel, indicating relatively uniform distributions of membrane polarization and induced EF within the ROI. The HD-tDCS with the transcranial channel had focused EF distributions under the transcranial channel and then induced EF might be decreased rapidly. Thus, we observed greater variation in membrane polarization in accordance with induced EF.

**Figure 3:**
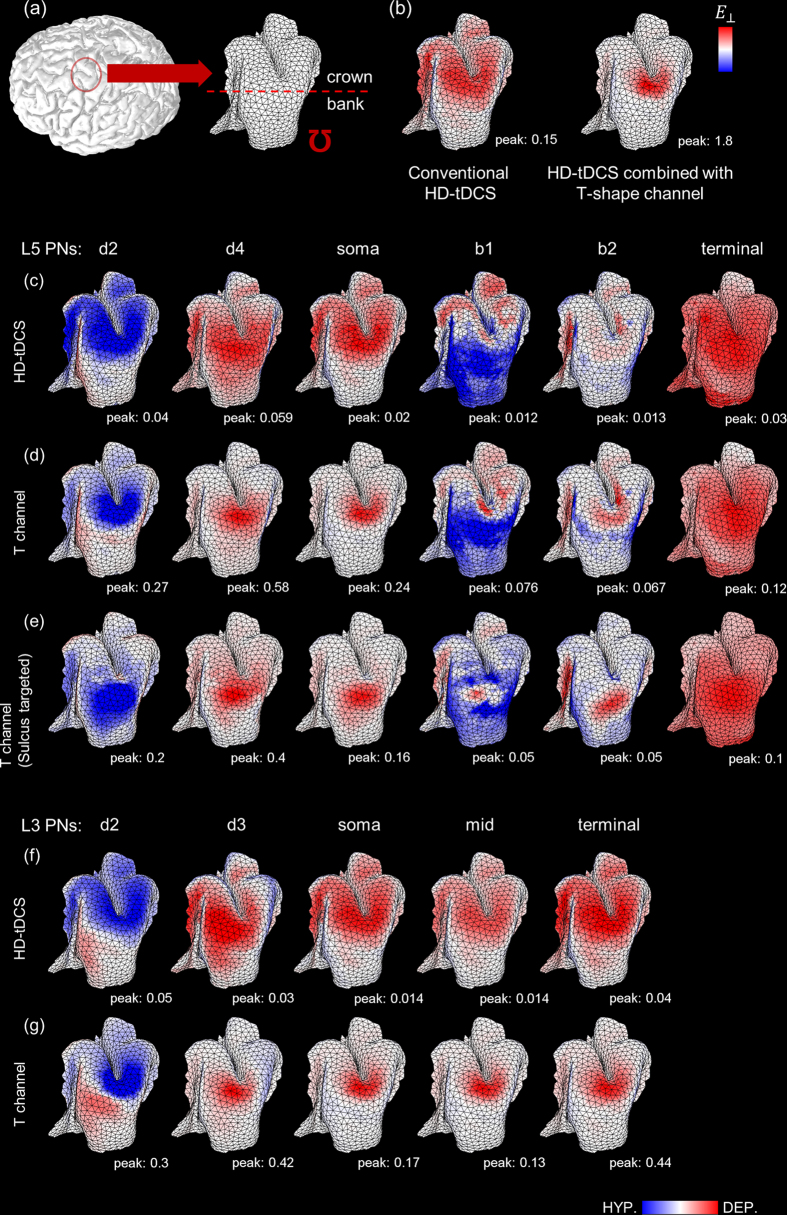
The spatial extent of membrane polarizations for L5 and L3 PNs focused on precentral gyrus. (**a**) We decoupled the target area which is the ohm-shaped hand knob, and the crown indicates the top of gyrus and the bank is the wall of the sulcus. We observed how the transcranial channel increases focality and intensity of the stimulus-induced membrane polarizations compared to conventional HD-tDCS without the T-shaped channel. We used two predicted neural responses of the EF strength perpendicular to the CSF-GM boundary (*E*_⊥_, (**b**)) and polarizations at specific compartments (**c–g**). Remarkably, the spatial distributions for perpendicular components of EFs resemble most polarization maps. Conventional HD-tDCS for L5 PNs (**c**) and L3 PNs (**f**) have quite diffused patterns of polarization compared to the HD-tDCS combined with the T-shaped channel for L5 PNs (**d**) and L3 PNs (**g**). (**e**) We further investigated the variations of polarization by shifting the target area from the top of gyrus to the sulcus. Color scales are adjusted according to peak values in white under each spatial extent.

For further observations, we visualized the spatial distributions of L5 and L3 PNs polarizations focused on the ohm-shaped hand knob (the target area, as illustrated in Fig. 3(a)). The T-shaped transcranial channel with 7 mm channel diameter was considered and compared to conventional HD-tDCS without the channel (Fig. 3). We compared the spatial extents of polarization with the magnitude of the radial component of EFs, because the stimulation might contribute to neural activation when the EFs are perpendicular to the cerebrospinal fluid (CSF) and GM boundary^24^,^25^,^26^. In Fig. 3(b), the positive value (red) indicates directed inward and thus most current flows through the top of the gyrus. The polarization maps for the dendritic trees, soma and axon initial segments (not shown but the axon initial segment shows consistent spatial distributions with the soma) also have similar patterns with the radial field. The upper parts of the dendrites (d2 for L5 and L3 PNs) were hyperpolarized while the lower parts of the dendrites (d4 for L5 PNs and d3 for L3 PNs) were depolarized. The axons for L5 PNs induced complex polarization patterns compared to the somatic polarizations. The L3 PNs have consistent polarization distributions for all compartments, which might be due to the restricted dimension of L3 PNs within GM.

Even though the transcranial channel increased the magnitude of induced EFs, the polarizations were restricted to the top of the hand knob and did not extend to the sulcus. To interpret this, we focused on how the transcranial channel affected the EF decay with the cortical depth by the distribution patterns of EFs on the cross-section view (Supplementary Fig. S2). We found that the transcranial channel could improve the magnitude of EFs but that the channel did not allow the EFs to flow deeper due to the narrow width of the sulcus (only slight changes were observed). We then shifted the electrodes and the channel to target the deeper area of the central sulcus. The highest field magnitudes were located at the curvature of the gyrus in both precentral and postcentral gyrus (Supplementary Fig. S3). By placing the transcranial channel to the sulcus, the stimulus-induced polarization (Fig. 3(e)) showed comparable peak values and similar but shifted polarization patterns compared to the results induced by targeting the top of the gyrus, and it could not reach the bottom of the sulcus (see Supplementary Fig. S4 for the simulations of L3 PNs).

**Figure 4:**
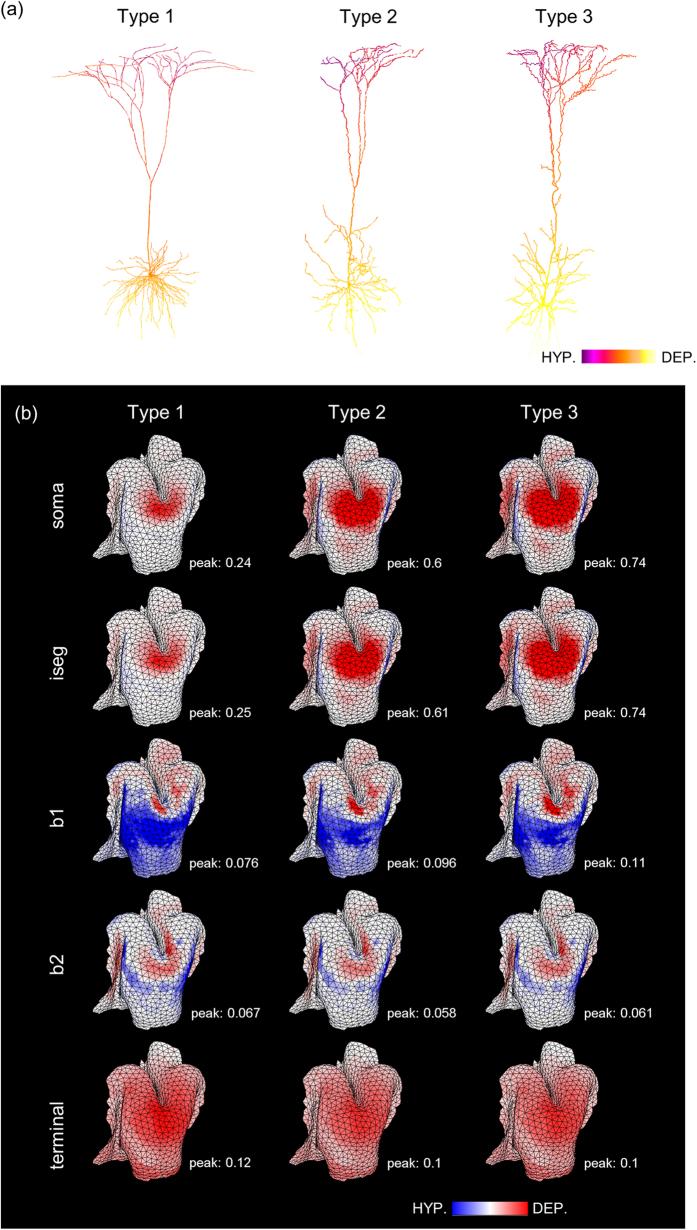
The impact of L5 PNs morphology on stimulus-induced membrane polarizations in the HD-tDCS combined with T-shaped transcranial channel. (**a**) Polarization profile illustrated with different morphologies; (**b**) The membrane polarization for L5 PNs focused on the precentral gyrus is measured at soma, axon initial segment (iseg), axon crossing boundary between GM and WM (b1), axon bending part (b2) and terminal; the color density scale is adjusted according to maximum value of the Type 1 PNs. Notable differences, according to different morphologies, are observed in soma and the axon initial segment with a higher magnitude of depolarization in type 2 and 3 PNs compared to type 1 PNs.

To investigate the role of neuronal morphology in determining the stimulus-induced membrane polarization, we incorporated two established L5 PNs additionally (type 2 and 3, as shown in Fig. 4(a)) that had different morphologies and same electrical properties were applied. We examined polarization profiles induced by HD-tDCS with T-shaped transcranial channel set to a diameter of 7 mm. Figure 4(a) shows polarization profiles of the dendritic trees of L5 PN directly under the transcranial channel. Similar polarization patterns were observed, with hyperpolarization observed in the upper parts of the dendrites corresponding to the dendrite depolarization positioned below the soma. Figure 4(b) illustrates the spatial distributions of polarization and the color scale is adjusted to the peak values induced by type 1 PNs for absolute comparison. The somata of type 2 and 3 PNs had 2.5–3 times higher peak values of polarizations compared to type 1 PNs and thus they induced somatic depolarization in wider regions (red in Fig. 4(b)). The magnitudes of axonal polarizations were slightly changed according to different PN morphologies but the spatial patterns did not alter. In addition, we found similar observations for the conventional HD-tDCS without the transcranial channel with increased somatic polarization with type 2 and 3 PNs compared to type 1 PNs.

Furthermore, we analyzed the cell-specific somatic coupling constant (mm) to define sub-threshold sensitivity, which is the somatic membrane polarization (mV) per unit EF applied (mV/mm)^19^,^20^,^23^. In the conventional HD-tDCS without the transcranial channel, the mean and standard deviations of the somatic coupling constant were 0.0784 ± 0.0384 in L5 PNs, and 0.0495 ± 0.0194 in L3 PNs, respectively. In the case of the transcranial channel, there were nominal variations in the coupling constant among the same models of PNs. For the L5 PNs that have different morphologies, the somatic coupling constants were 0.0416 ± 0.1667 for type 2 and 0.0673 ± 0.2031 for type 3 PNs. The coupling constant was within the experimental range in Radman *et al*.^20^, and we found similar observations, in that the different morphologies of the PNs caused the different coupling constant. The notable morphological difference was that, compared to type 1 PNs, type 2 and 3 PNs had flat dendritic trees. When the somatic diameter was increased, the somatic polarization was decreased (Supplementary Fig. S5). This agrees with other modeling studies^19^,^27^,^28^ showing that different morphologies of PNs play a critical role in determining neural polarization. As shown in Fig. 4(b), we could estimate the consistent characteristics of polarization patterns regardless of varying morphology, such as those parts that might be depolarized or hyperpolarized.

### Displacement of the electrodes from the transcranial channel

Aligning the active electrode with the transcranial channel is difficult; even a small shift in the electrode may produce a substantial reduction in the stimulation effect in the target area. Therefore, it is essential to identify an acceptable range of electrode displacements relative to the transcranial channel. Accordingly, we investigated the influence of the displacement of the stimulus electrodes, while we fixed the transcranial channel to the target area. We focused on the T-shaped channel at 7 mm diameter, since it showed the most efficient stimulation effect.

**Figure 5:**
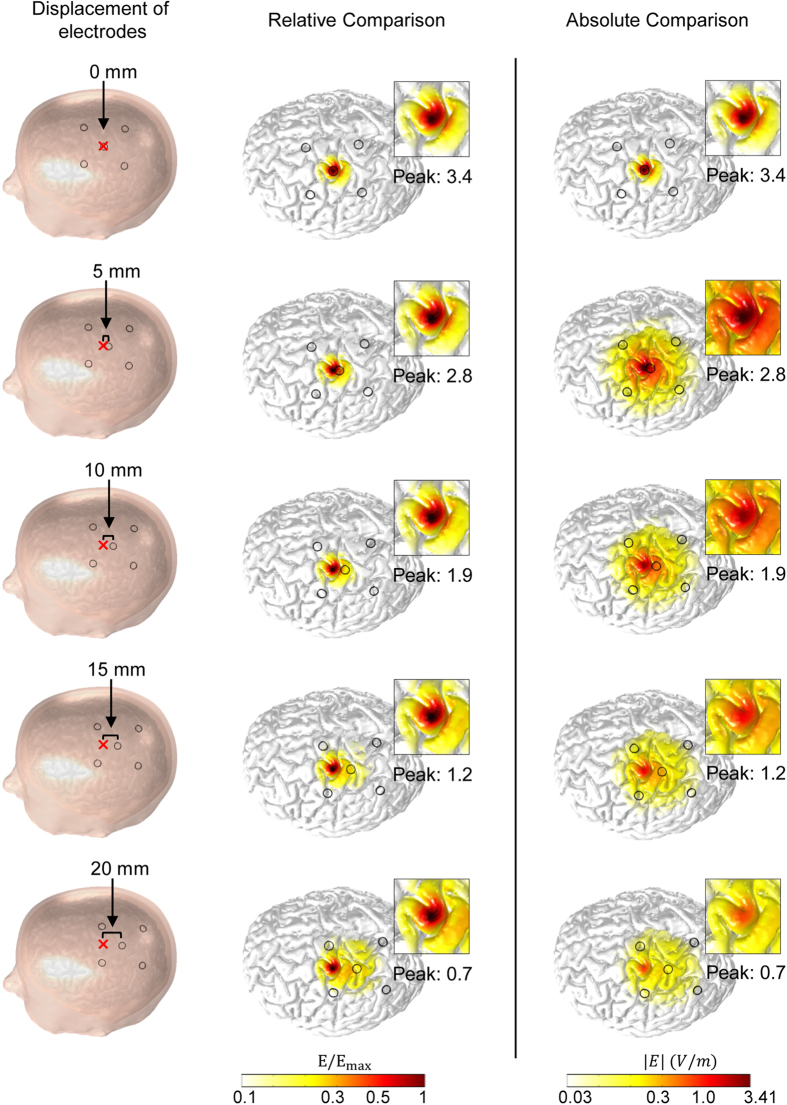
EF distributions with respect to displacement of the center (active) electrode relative to the transcranial channel. In the 1st column, ‘x’ indicates the fixed location of the transcranial channel that targets the ROI, and ‘o’ represents electrodes positioned in a circular fashion with the center electrode surrounded by four return electrodes. Here, a T-shaped channel was used. In the 2nd column, the color scales were adjusted to the peak value of EFs, while in the 3rd column, the color scale was fixed to the EF peak value with no displacement.

Figure 5 presents the stimulus-induced EF distributions; the displacement of the center (active) electrode relative to the transcranial channel increased in increments of 5 mm and induced gradually decreasing peak EF values, as well as more diffuse EF distributions. Displacements of up to 5 mm maintained a relatively higher peak EF on the targeted hand knob; however, when the displacement reached approximately 20 mm, the EF magnitude decreased by up to 20% compared to the case with no displacement, and far more extensive spatial EF distributions were observed. Even the induced EF strength decreased as the displacement of the electrodes increased, and we found that the target area was stimulated consistently. Because stimulus-induced current flowed to the transcranial channel, it seemed to reach the target area even when the electrodes were located far from that area.

**Figure 6:**
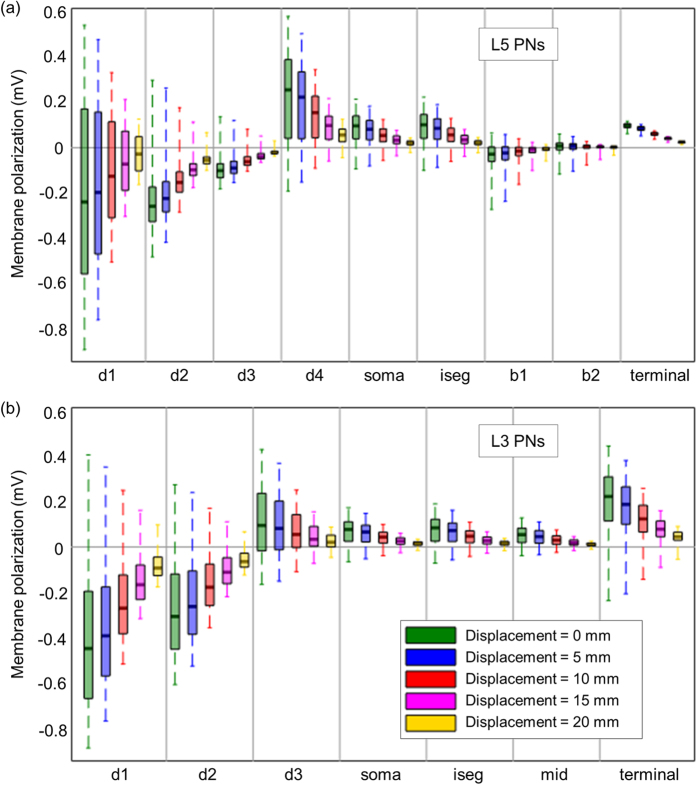
The distributions of membrane polarization in neuronal compartments (ROI) in L5 (**a**) and L3 PNs (**b**). Membrane polarizations of PNs was analyzed in the region of interest (ROI) with 5 mm radius in the middle of the hand knob, where it is directly under the center electrode, and thus total 112 numbers of L5 and L3 PNs were analyzed. Then, displacement of the center electrode relative to the transcranial channel varied in increments of 5 mm. The median and the first and third quartiles of membrane polarization of PNs in the ROI are illustrated; whiskers indicate maximum and minimum membrane polarizations in the compartments.

As expected, the maximum value of the membrane polarization decreased in both L5 and L3 PNs (Fig. 6) as the displacement of the center electrode from the transcranial channel increased. When the displacement of the center electrode was up to 5 mm in the postcentral gyrus, we observed that the peak somatic polarization dropped by up to 87% compared to no displacement (exact match between the center electrode and the transcranial channel). Furthermore, when the displacement increased to 10 mm, membrane polarizations in all compartments dropped to 60% or lower.

## Discussion

The transcranial channel resembles an epidural peg or screw electrodes, which are used for intracranial recordings to map seizure foci^29^,^30^,^31^,^32^. The epidural peg electrodes are flexible and mushroom-shaped, while the screw electrodes are tapered, and can be inserted easily and removed percutaneously. Similar to the epidural peg and screw electrodes, the transcranial channel has the advantages, in that it does not require dissection of the epidural space, craniotomy, or a larger bur hole compared to the epidural strip. Therefore, it has lower risks of infection, hemorrhage and has enhanced patient comfort. Moreover, when the treatment is altered, such as a change in technique or target area, the implanted transcranial channel can be removed in the same way epidural pegs or screw electrodes are removed. Because the epidural screw electrode might permit easier insertion and removal compared to peg electrodes, which require twist-drilled skull holes^31^,^32^, the improved design of the tapered screw for the transcranial channel should be considered. Another potential use of the transcranial channel might be to deliver electrical brain activity to measure electroencephalography (EEG), which could alleviate the fundamental limitation of a blurred signal attributable to the preferential flow of electrical current induced by brain activity through the intervening tissue.

Transcranial direct current stimulation (tDCS), which delivers a weak direct current (up to 2 mA) through electrodes on the scalp, has attracted considerable interest, as it is safe, inexpensive, portable, and simple to implement^4^,^5^,^6^,^7^. It conveys a weak current that activates a relatively larger brain area, even in areas under the target electrode^1^,^8^,^10^, as most of the current applied is shunted because of the high impedance of the scalp and skull dispersion. Recently, Datta *et al*.^8^,^9^ proposed HD-tDCS and showed that it results in greater targeted brain modulation compared to conventional tDCS, as the electrode montage of tDCS can shape the induced current distribution^6^,^9^,^33^,^34^. They revealed that it induces the maximal magnitude of the stimulus-induced EF directly beneath the active electrode, and enhanced spatial focality compared to the conventional tDCS. However, HD-tDCS induced EF distributions on the brain are relatively widespread compared to an invasive approach, because intermediate tissues (e.g., scalp, skull, CSF) disturb the current flow produced by HD-tDCS, while an invasive approach has no obstacles. To achieve neuromodulation that is targeted with minimal invasiveness, we introduced HD-tDCS combined with the transcranial channel. It improves stimulus efficacy in terms of higher intensity and focality; however, as far as we know, it has been proposed only conceptually and its effects have not been investigated. Therefore, our computational study demonstrated the ways in which the transcranial channel improves the efficiency of stimulation effects.

As the transcranial channel has fewer shunting effects and conveys injected current to the brain through the relatively highly conductive material, it has greater stimulus efficiency at the target area, as is shown by the stimulus-induced EF distributions between the conventional HD-tDCS with and without the transcranial channel (Fig. 1). While most previous computational studies have investigated the model leverage in terms of EF intensity based on the assumption that such EF is correlated with the physiological changes desired^4^,^9^,^35^,^36^, the neural excitability is affected not only by the magnitude of EF, but also by the orientation to the cortical neurons and their morphology^19^,^20^,^37^. Thus, we further investigated the response of variable relative polarization in each compartment of L5 and L3 PNs by including the transcranial channel. The general neuronal responses were hyperpolarization of the upper part of the dendrites and depolarization in the other compartments. This is consistent with the response of PNs to radially-directed uniform EF^19^,^20^, because the modeled PNs were located on the top of the gyrus perpendicular to the cortex, and thus the extracellular fields to the PNs flowed predominantly perpendicular to the cortical surface. The membrane polarization in the conventional HD-tDCS model without the transcranial channel had a relatively smaller variation within the ROI, while HD-tDCS with the transcranial channel increased it. This indicates that, in accordance with EF distributions, the transcranial channel delivered a focused stimulus to the PNs in ROI compared to the conventional HD-tDCS without the transcranial channel.

We inspected the EFs that were decomposed into radial and tangential components (Fig. 1) and estimated what components of the induced EFs contributed to modulating the polarizations of PNs. We found that PNs were preferentially polarized by radial field (Fig. 3); this is because radial field is in the direction of the primary axis of PNs within the GM that were oriented perpendicular to the cortical surface. Therefore, we could extrapolate the neural polarization patterns using the radial component of EFs; however, there were some exceptions in the axon of L5 PN. The axon of L5 PN showed inconsistent patterns with the spatial extent of radial field; this is because the axon of L3 PN was located within the GM, however, the axon of L5 PN was further extended to the WM; the axon of L5 PN after crossing the interface between the GM and WM had different directionality to radial field, as shown in Fig. 2(a).

The PNs showed polarization in a compartment-specific manner in that they were hyperpolarized at dendrites in the superficial cortex, and were depolarized at both soma and axon initial segment. Somatic polarization may be associated with spontaneous activity^22^,^38^ and synaptic efficacy^39^,^40^. Dendrites and axon may play a significant role in the somatic polarization^41^. Thus, we observed that somata depolarization varied depending on dendritic morphology (Fig. 4). Furthermore, polarization of the dendrites and axon may influence synaptic processing^23^. Therefore, it is necessary to quantify compartment-specific responses to induced EF.

In this study, we demonstrated that the T-type channel with a 7 mm diameter was the most effective approach. A reasonably acceptable displacement range between the stimulus electrode and the transcranial channel was 5 mm, since it produced higher than 80% performance compared to the case in which there was no displacement. Although the displacement of the active electrode reached 20 mm, we observed that the membrane polarization induced by the inclusion of the transcranial channel was higher than that of the conventional HD-tDCS without the transcranial channel. In addition, substrates around the transcranial channel were believed to reduce shunting of the current (not shown here); however, this yielded a slightly reduced polarization, and we observed no substantial difference compared to the case without substrates.

The enhanced intensity and focality of EF by combining tDCS and the transcranial channel can be expected in the study from Datta *et al*., who investigated the influence of skull defects and skull plates, having higher conductivity relative to the skull on tDCS induced EF^14^. They found that the defects altered the intensity and location of current flow and suggested that the higher conductive materials might be beneficial in targeting brain regions. The study of Datta *et al*. was proposed to find the changes of the overall current flow patterns by the presence of skull defects or skull plates. However, we focused how to maximize both EFs and neural polarizations by higher conductive materials that act as a preferential pathway. We simulated not only the realistic EF distributions but also neural responses using the compartmental models of PNs, and found different polarization patterns at specific neural compartment that cannot be estimated by induced EF. Furthermore, we investigated the dimension of the transcranial channel and the reasonable range of electrode displacement that maximizes neural polarizations.

In accordance with the safety issues of the study from Datta *et al*., the increased magnitude of induced EF due to high conductive materials might cause potential safety concerns^14^. The concentration of the induced current can be predicted, and we could control the magnitude of the injected current. In addition, according to Wingeier *et al*., who proposed the conceptual models of the transcranial channel, a heat absorbing or cooling device might be combined to reduce the induced heat when a temperature exceeds a predetermined limit^11^. In addition, we may expect possible electrochemical reactions at the tissue-channel interface through the tissue-electrode interface. To transfer the external current into the brain area targeted, the electric current flows into surrounding tissue and is converted into ion movement. Then, some undesirable reactions may be induced, for example, production of possible toxic substances and denaturation of proteins. This is often referred to faradaic current, which is absorbed by a reversed electrochemical reaction by tailing phase of stimulus and by controlling the charge passes per phase in order to minimize the faradaic current^11^. The proposed modeling results presented a preliminary study for the use of the transcranial channel; thus, further investigation on potential safety concerns should be done prior to clinical application. The present study is a first step towards understanding the basis of how the transcranial channel helps to convey the external current to the brain effectively and the underlying mechanisms of cellular responses.

The transcranial channel, combined with HD-tDCS, is proposed for better targeting with reduced shunting effects and provides a stable procedure by fixing maximum excitability at the target area, because the transcranial channel is implanted. Further, applying multiple transcranial channels could tailor the stimulus-induced polarization with respect to specific target area. Therefore, when the transcranial channel is used with repetitive tDCS, it may help to induce prolonged, stabilized and lasting effect and to treat a variety of neurological disorders. Particularly, the transcranial channel may be beneficial for rehabilitation for strokes that are caused by motor defect and focal epileptic cortex, because the transcranial channel restricts its effects to the focal target area.

The accuracy of the dimensions and conductivity values incorporated in the volume conduction head model may be a dominant factor in computational studies^4^,^8^,^18^. In this study, we applied anatomically realistic head geometry derived from MRI data, but the dura mater was constructed arbitrarily by dilating the boundary between the CSF and skull layer. According to the normal anatomical model section of the BrainWeb Simulated Brain Database^42^, the skull layer includes the dura mater generally, but when the dura is thin, it could be included in the CSF layer due to a partial volume effect. To test the effects of dura mater, we compared the conventional HD-tDCS model with and without dura mater. The Model with the dura mater increased the induced EF strength, EF peak, and somatic polarization slightly, but changing the dura mater by shrinking or dilating the boundary between the CSF/skull did not alter the results substantially. We used the computational head model assigned to isotropic conductivity. The effects of anisotropic conductivity in brain stimulation have been addressed widely^43^,^44^,^45^,^46^,^47^, and Suh *et al*. reported that there is a significant effect on stimulus-induced EF that is dependent on anisotropic skull conductivity. However, Shahid *et al*. suggested that anisotropy does not modulate significant changes in comparisons across montages. Therefore, although this work includes inherent modeling limitations as a preliminary study, it may increase our understanding of the ways in which the transcranial channel coupled with HD-tDCS affect cellular modulation.

Stimulation with the conventional tDCS that induces a broadly distributed EF might be desirable when the target area is defined poorly^8^,^36^. However, recent research has addressed the ability of focal stimulation to achieve more refined targeting of neuromodulation^5^,^6^,^9^,^10^,^33^,^36^,^48^,^49^,^50^,^51^,^52^, because the increased focality of stimulation might improve the functional interpretation of stimulation effects, as the outcomes of brain stimulation are linked to the target area. Furthermore, once the target areas are determined, targeted neuromodulation might be paramount and avoid unwanted effects in other brain areas. To investigate the influence of the focal stimulation, Kuo *et al*.^10^ compared the conventional tDCS with HD-tDCS in a neurophysiological study and found a delayed peak of excitability with longer lasting after-effects after HD-tDCS compared to the conventional tDCS; this might be due to the differential modulation of different groups of neurons. Clearly, there are numerous types of neuronal models that may be related to therapeutic tDCS results. Therefore, this computational study should be extended to other kinds of neuronal models in order to understand the underlying mechanisms of the focality effects.

## Methods

### The volume conduction model of an anatomically realistic head

**Figure 7:**
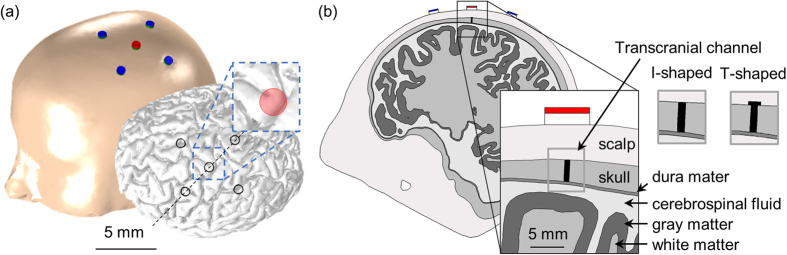
The shape of the anatomically realistic head model. Electrodes were placed (**a**) overlaying the head (left) and brain (right). The red circle in the right inset represents the ROI on the hand knob. The cross-sectional view along the black dotted line is illustrated (**b**) and shows the two types of transcranial channels.

We developed a volume conduction model of an anatomically realistic head for HD-tDCS using human magnetic resonance imaging (MRI) acquired from SimNIBS^46^ (Fig. 7(a)). We note that this human MRI data is anonymous and publicly accessible. For this reason, this study did not require Institutional Review Board (IRB) approval from the Gwangju Institute of Science and Technology (GIST). The model was segmented by the white matter (WM), gray matter (GM), cerebrospinal fluid (CSF), skull, and scalp, respectively. Dura mater was made artificially by deleting 0.5 mm^53^ of the boundary between the CSF and skull layer based on the assumption that it was mapped to the skull layer^42^,^54^. Then, we constructed disc-type electrodes (height = 1 mm; radius = 4 mm) to form a 4 × 1 HD-tDCS electrode montage with the “active” center electrode on the target hand knob in the precentral gyrus^55^, and with four “return” electrodes positioned in a circular fashion^8^. CCNY-4 gel (height = 2 mm; radius = 4 mm) was modeled between the scalp and the electrodes. Thereafter, two types of transcranial channels were designed, one an I-shaped channel with a shaft only, and the other a T-shaped channel that consisted of a shaft with a hat. These transcranial channels were designed to traverse the entire thickness of the skull (Fig. 7(b)). The diameters of the shaft varied from 1 to 9 mm and we set the diameter of the hat to the shaft diameter +1 mm.

3D-optimized tetrahedral volume mesh was constructed from the surface mesh using iso2mesh^56^ and tetgen^57^. There were approximately 18 million total tetrahedral elements, with far smaller elements distributed around the electrode, transcranial channel, and regions of interest (ROIs) in the precentral gyrus; element size ranged from an average of 0.0043 mm^3^ (in the transcranial channel) to an average of 1.25 mm^3^ (in the skull). The electrical conductivities of the compartments were set to isotropic values (in units of S/m)^35^,^58^,^59^,^60^: scalp, 0.465; skull, 0.01; dura mater, 0.065; CSF, 1.65; GM, 0.276; WM, 0.126; gel, 0.30, and electrode, 5.80e^7^. In this paper, we considered the transcranial channel made of titanium with the conductivity of 7.40e^5^ S/m, because it is a biocompatible material used commonly as a skull plate^14^. The Laplace equation was solved with the boundary conditions—the exposed surface of the active electrode was set to 1 mA inward current, those of the return electrodes were set to ground, and all other external boundaries were set to be insulated. We used the linear system solver of the conjugate gradient method with a relative tolerance of 1 × 10^−9^ in COMSOL Multiphysics 5.2 (Comsol Inc, Massachusetts).

### The compartmental model of pyramidal neurons

We constructed two kinds of compartmental models, L5 and L3 PNs. Their morphology and electrical properties were taken from the cat visual cortex^61^. Furthermore, to investigate the robustness of the estimated polarizations for the neuronal morphology, we adapted two established L5 PNs additionally that were composed by different morphologies (illustrated in Fig. 4(a))^62^,^63^. The same compartment composed of axons and electrical properties were applied. The PNs were lengthened following the irregular dimensions of the cortex to reach the dendritic trees to layer 1^58^,^64^. Simulations for the compartmental models of PNs were performed in the NEURON environment^65^.

L5 and L3 PNs were coupled indirectly within the hand knob (Fig. 7(a)) in the anatomically realistic head model (for details, see Seo *et al*.^45^,^66^). Because PNs are oriented perpendicular to the cortex^67^,^68^,^69^, we set the neuronal orientation perpendicular to the brain surface, with boundary elements between CSF and GM, and the axons of L5 PNs were curved beyond the boundary between the GM and WM^45^,^58^,^59^,^70^. We constructed L5 and L3 PNs (2137 numbers of PNs each), and then applied the stimulus-induced potential fields distributed in the anatomically realistic head model at each center point of the compartmental models of PNs by extracellular stimulation. As complex membrane polarization was evoked according to neuronal morphology and orientation relative to the induced EF^19^,^20^,^23^, we investigated a range of steady-state membrane polarizations in each compartment (dendrites, soma, initial segment, and axons) of the L5 and L3 PNs. To investigate the focality and intensity of the target area we analyzed the median value of polarization and their deviations in pre-selected ROI, where it is directly under the center electrode, as shown by the red circle in Fig. 7(a). This process was implemented in MATLAB (MathWorks, Natick, MA, USA) and COMSOL 5.2 with MATLAB.

## Additional Information

**How to cite this article**: Seo, H. *et al*. The Effect of a Transcranial Channel as a Skull/Brain Interface in High-Definition Transcranial Direct Current Stimulation—A Computational Study. *Sci. Rep.* **7**, 40612; doi: 10.1038/srep40612 (2017).

**Publisher's note:** Springer Nature remains neutral with regard to jurisdictional claims in published maps and institutional affiliations.

## Supplementary Material

Supplementary Information

## Figures and Tables

**Table 1:** Variation in the stimulus-induced EF within the brain with increments in the shaft diameter of the T-shaped and I-shaped channels.

Channel Type	Shaft diameter	Peak EF (V/m)	Vol_50_	Vol_60_	Vol_70_	Vol_80_
Conventional HD-tDCS		0.30	1538.20	366.99	19.74	0.17
T-shaped Channel	1 mm	0.99	12.02	5.02	2.05	0.54
	3 mm	2.53	12.78	5.78	2.42	0.70
	5 mm	3.35	22.81	10.19	3.78	0.87
	7 mm	3.40	52.98	23.87	8.69	1.76
	9 mm	3.09	92.19	41.11	16.68	5.27
I-shaped Channel	1 mm	0.88	13.49	5.45	2.22	0.58
	3 mm	2.28	13.04	5.88	2.46	0.70
	5 mm	3.10	23.04	10.28	3.79	0.88
	7 mm	3.26	53.22	23.92	8.71	1.76
	9 mm	2.97	92.60	41.23	16.72	5.29

The conventional HD-tDCS without the transcranial channel is compared. The peak EF magnitude (V/m) and cortex volumes in excess of 50%, 60%, 70%, or 80% of the peak EF magnitude (Vol_50_, Vol_60_, Vol_70_ and Vol_80_ (mm^3^)) were observed. Note the conventional HD-tDCS has a lower peak EF value and lower relative focality compared to that with the transcranial channel.
