# Supplementary material for: The Effect of a Transcranial Channel as a Skull/Brain Interface in High-Definition Transcranial Direct Current Stimulation—A Computational Study
Source: Sci Rep. 2017 Jan 13;7:40612. doi: 10.1038/srep40612 (PMC5233984; doi:10.1038/srep40612)
Supplement: Supplementary Information [file srep40612-s1.pdf]

The Effect of a Transcranial Channel as a Skull/Brain Interface in High-Definition Transcranial Direct Current Stimulation—A Computational Study

Hyeon Seo[1], Hyoung-Ihl Kim[2], and Sung Chan Jun[1*]

[1]Gwangju Institute of Science & Technology, School of Electrical Engineering and Computer Science, Gwangju, 61005, South Korea

[2]Gwangju Institute of Science & Technology, Department of Biomedical Science and Engineering, Gwangju, 61005, South Korea

[*]scjun@gist.ac.kr

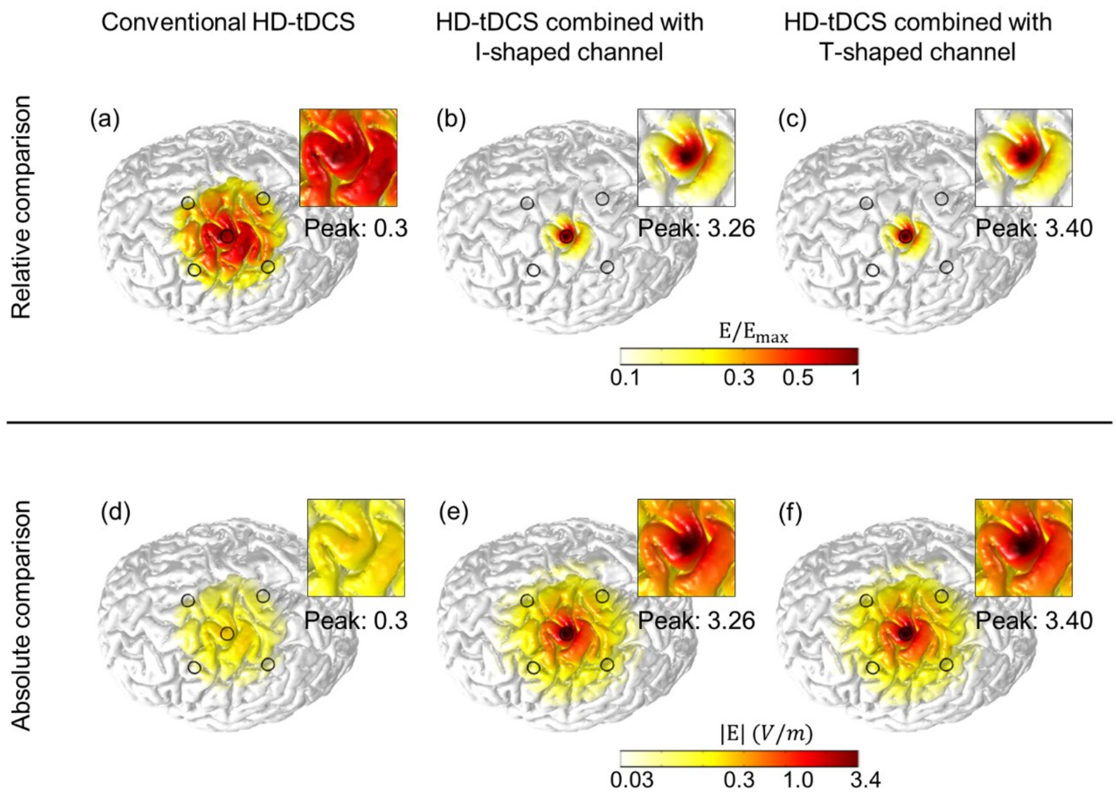

Supplementary Figure S1. Distribution patterns of stimulus-induced EFs on the cortical surface. Comparison of the conventional HD-tDCS without the transcranial channel (a and d) and with implantations of the I- (b and e) and T- shaped transcranial channels at the channel diameter of 7 mm (c and f); the color density scale is adjusted according to the maximum value of the EFs (a)-(c), and the color density is rescaled to the maximum value of the EF-induced by the T-shaped channel (d)-(f) under the montages considered.

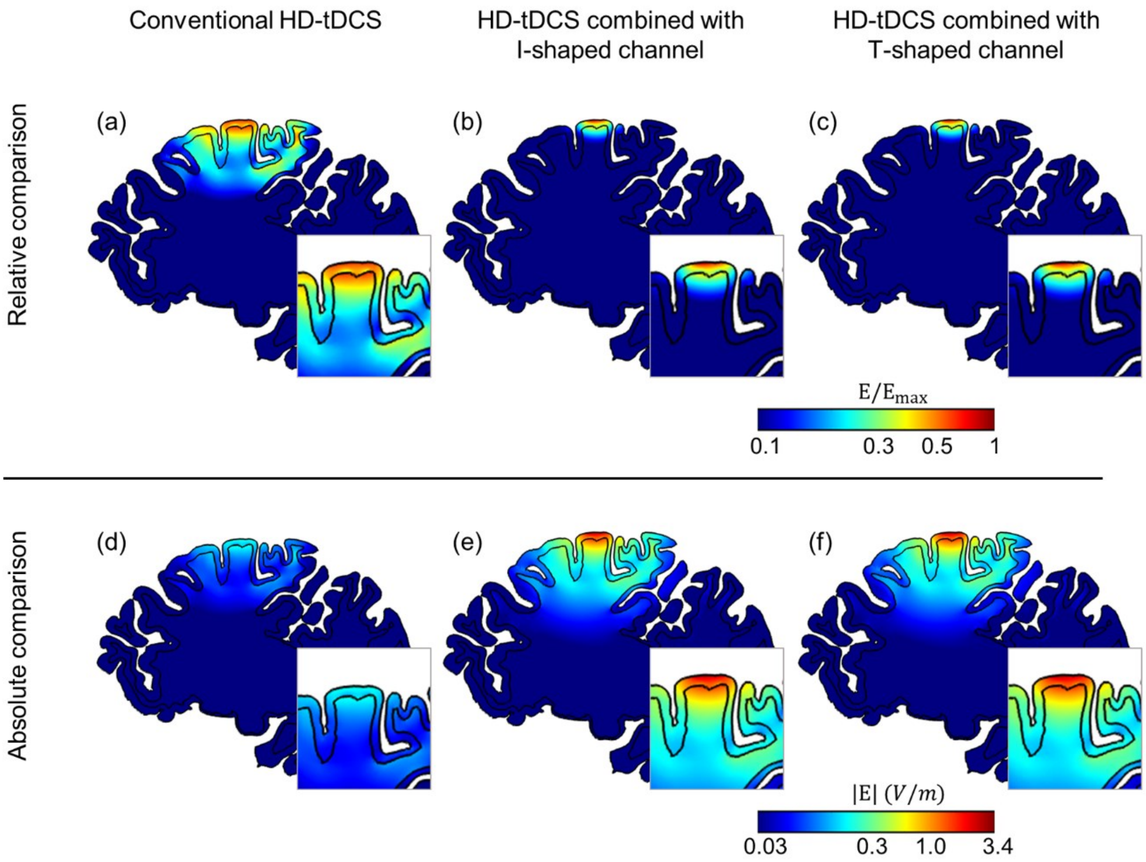

Supplementary Figure S2. Distribution patterns of stimulus-induced electric fields on cross-sectional view. In (a)-(c), the color density scale is normalized to each maximum value of electric fields. HD-tDCS with the transcranial channel induces higher electric fields focalized on the top of gyrus, while conventional HD-tDCS without the transcranial channel has diffused patterns of electric fields. In (d)-(f), scale is adjusted to the maximum value of the electric fields induced by the T-shaped transcranial channel. The transcranial channel conveys the higher strength of stimulus-induced electric fields to the brain by reducing the shunting currents compared to the conventional HD-tDCS.

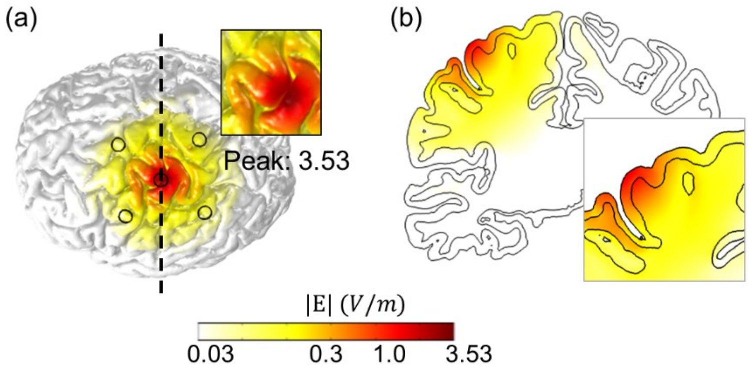

Supplementary Figure S3. Distribution patterns of stimulus-induced electric fields when the electrodes and the T-shaped channel are placed above the central sulcus. Higher strength of electric fields is focused on the peripheral region of the sulcus in cortical surface view (a) and sulcal wall in cross-section view (b).

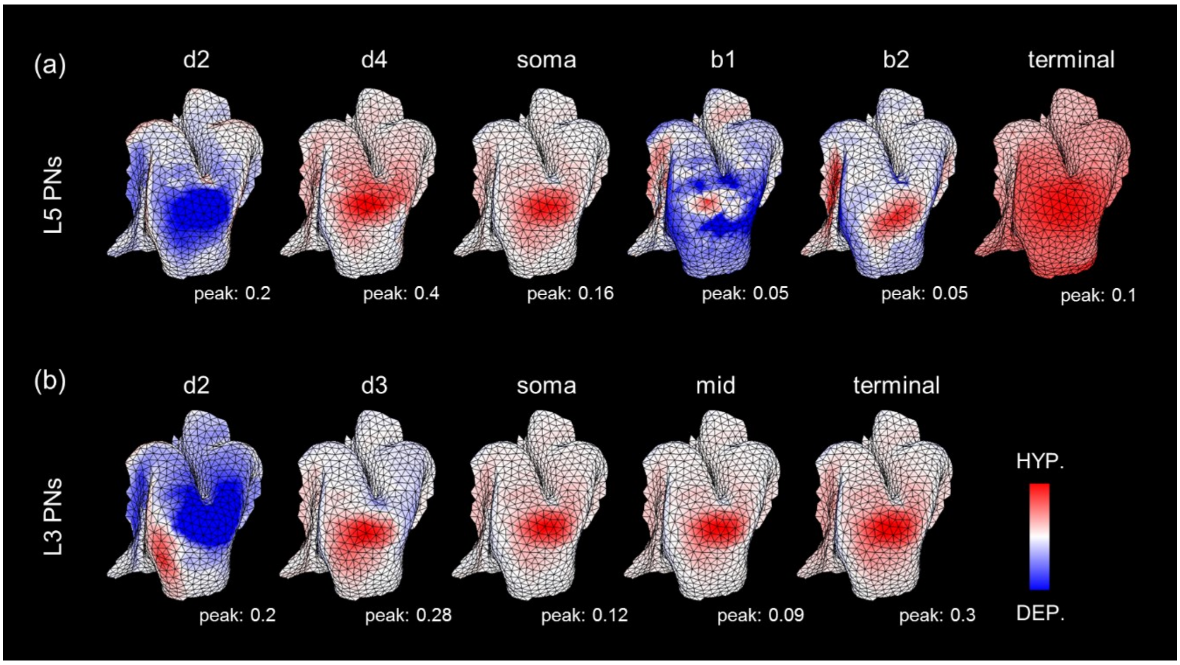

Supplementary Figure S4. The spatial extent of membrane polarization for L5 (a) and L3 (b) PNs when the electrodes and T-shaped channel are placed above the central sulcus. PNs in the lip and upper part of bank along the sulcal wall are polarized.

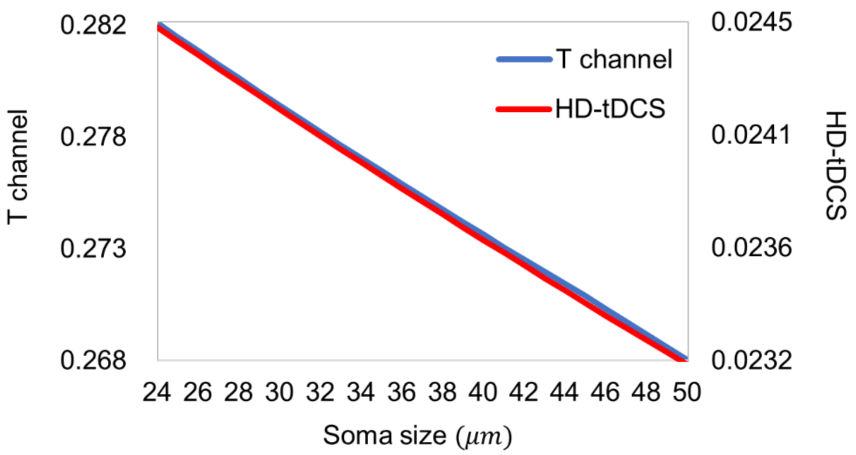

Supplementary Figure S5. The relationship between somatic size and stimulus-induced polarization. The membrane polarizations as a function of somatic size in both HD-tDCS with and without transcranial channel.
